# Cytoprotective Effect of the UCP2-SIRT3 Signaling Pathway by Decreasing Mitochondrial Oxidative Stress on Cerebral Ischemia–Reperfusion Injury

**DOI:** 10.3390/ijms18071599

**Published:** 2017-07-24

**Authors:** Jing Su, Jie Liu, Xiao-Yu Yan, Yong Zhang, Juan-Juan Zhang, Li-Chao Zhang, Lian-Kun Sun

**Affiliations:** Key Laboratory of Pathobiology, Ministry of Education, Department of Pathophysiology, College of Basic Medical Sciences, Jilin University, 126 Xinmin Street, Changchun 130021, China; sujing@jlu.edu.cn (J.S.); lsl20062007@163.com (J.L.); yanxiaoyu0713@163.com (X.-Y.Y.); zyloulan@163.com (Y.Z.); zhangjuanjuan8422@163.com (J.-J.Z.); zhanglichao3468@163.com (L.-C.Z.)

**Keywords:** uncoupling protein 2 (UCP2), sirtuin 3 (SIRT3), peroxisome proliferator-activated receptor gamma coactivator 1-alpha (PGC1α), oxidative stress, cerebral ischemia/reperfusion injury, mitochondria

## Abstract

Recovered blood supply after cerebral ischemia for a certain period of time fails to restore brain function, with more severe dysfunctional problems developing, called cerebral ischemia–reperfusion injury (CIR). CIR involves several extremely complex pathophysiological processes in which the interactions between key factors at various stages have not been fully elucidated. Mitochondrial dysfunction is one of the most important mechanisms of CIR. The mitochondrial deacetylase, sirtuin 3 (SIRT3), can inhibit mitochondrial oxidative stress by deacetylation, to maintain mitochondrial stability. Uncoupling protein 2 (UCP2) regulates ATP (Adenosine triphosphate) and reactive oxygen species production by affecting the mitochondrial respiratory chain, which may play a protective role in CIR. Finally, we propose that UCP2 regulates the activity of SIRT3 through sensing the energy level and, in turn, maintaining the mitochondrial steady state, which demonstrates a cytoprotective effect on CIR.

## 1. Introduction

The brain is the most sensitive organ to hypoxia. Previous studies have shown that the damage caused by brief ischemia and hypoxia in brain tissue is reversible, whereas longer duration causes irreversible brain damage, resulting in dysfunctional tissues and organs throughout the body [1,2]. Therefore, a timely and effective recovery of cerebral blood flow is critical for the clinical treatment of cerebral ischemic diseases. However, the restoration of perfusion contributes to the deterioration of the damage, called an ischemia–reperfusion injury, which complicates clinical treatment [3].

Mitochondria are the regulation centers of cell energy metabolism and oxidative stress. During cerebral ischemia–reperfusion, the electron transfer is inhibited in the dysfunctional mitochondria, increasing the proton leak and reactive oxygen species (ROS) generation. Furthermore, the mitochondria are the main target of ROS, which further aggravates the mitochondrial damage, leading to cell energy metabolism disorders [4]. Concomitantly, mitochondria are at the center of cellular communication networks, and various mitochondria-associated signaling pathways may lead to cell death [5,6]. Thus, prevention of mitochondrial damage is important for decreasing the harmful effects of ischemia–reperfusion injury on nerve cells.

Sirtuin 3 (SIRT3) is the main mitochondrial NAD^+^-dependent deacetylase. It is also a member of the sirtuin family, which protects mitochondria from damage [7]. SIRT3 regulates the expression and activation of mitochondrial proteins, decreases ROS production and plays a key role in mitochondrial adaptation and stress response [8]. Previous studies have suggested that SIRT3 is highly expressed in Alzheimer’s disease and aging cells [9,10]. Another experiment with primary neurons indicated that SIRT3 increased with the increase in ROS production [11]. Furthermore, it has been revealed that SIRT3 overexpression reduced mitochondrial damage and cell death in a muscular model of amyotrophic lateral sclerosis [12]. However, the role of SIRT3 in the mitochondrial protection mechanism is not fully understood. Therefore, in this review we tried to elucidate the pathway(s) involved in the mitochondrial protection mechanism by integrating a variety of proteins associated with SIRT3. This study may provide new strategies for cerebral ischemia–reperfusion injury therapy.

## 2. Mitochondrial Dysfunction and Reperfusion Injury

**Figure 1:**
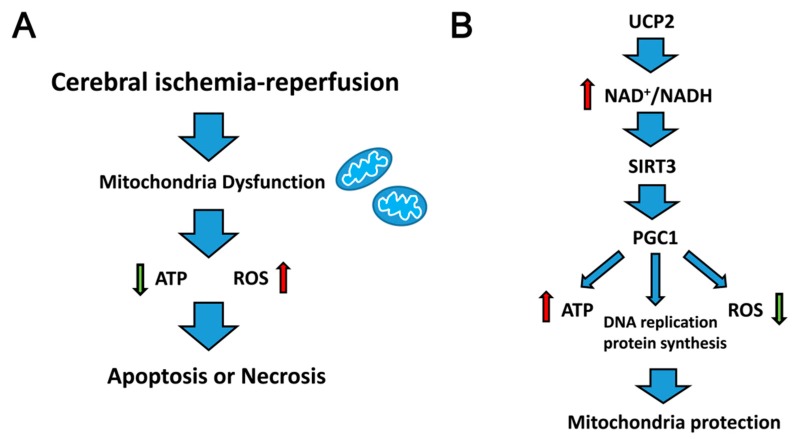
Proposed model for UCP2-SIRT3 (uncoupling protein 2-sirtuin 3) signaling pathway on cerebral ischemia–reperfusion injury. (**A**) Mitochondrial dysfunction caused by cerebral ischemia–reperfusion injury leads cells to death. (**B**) Protective mechanism of UCP2-SIRT3 signaling pathway on mitochondria damage.

Cerebral ischemia–reperfusion injury is a complex pathological process, including oxidative stress mediated by increased ROS, calcium overload caused by increased intracellular Ca^2+^ flow along with reduced discharge, and inflammation, which causes the aggregation of white blood cells [4,13,14]. These mechanisms co-exist and cooperate with each other, forming a vicious cycle that eventually leads to cell apoptosis or necrosis. Several studies have indicated that mitochondrial dysfunction first appears during acute ischemia and anoxia. During this condition mitochondria showed increased volume, edema, broken and dissolved cristae, ruptured membranes and decreased quantity [15]. Impaired mitochondrial function directly affects cell survival. First, increased ROS induces protein, lecithin, and DNA damage. Second, the cell energy supplement barrier contributes to an imbalance in metabolites and ion transport, resulting in intracellular calcium overload. Finally, mitochondria biosynthesis and related molecular defects cause the accumulation of lipids and metabolites in the cell [16]. Recently, accumulating evidence has suggested that mitochondrial damage plays a key role in cell death caused by ischemia and anoxia [17,18]. Therefore, it would be helpful to promote nerve cell survival through mitochondrial protection. It has been reported that antioxidants protect complexes I–III, thus strengthening the respiratory function in acute stroke, in which ATP production is increased and the ischemia-induced ATP decrease is temporized [19]. It is beneficial to minimize cell damage and promote nerve cell survival. Therefore, researching the mechanism of mitochondrial protection may provide an important breakthrough in reinjection therapy (Figure 1A).

## 3. Sirtuin 3 (SIRT3) and Mitochondrial Protection 

SIRT3 is a member of the NAD^+^-dependent deacetylase sirtuin family, which appears to monitor and direct the integrity of cellular metabolism proteins [12]. SIRT3 is mainly distributed in the kidney, brain, heart and liver, and in other tissues and organs that are rich in mitochondria [20]. Initially, SIRT3 is translated into an inactive precursor in the cytoplasm. When it is transferred into the mitochondria, it is processed by mitochondrial processing peptidase (MPP) and losses in 101 amino acids at the N-terminal. The activated form can interact with two types of substrate: the first one is proteins or peptides containing an acetylated lysine, and the other is NAD^+^ [21]. SIRT3 first interacts with an acetylated protein, forming a deacetylation site. Then, the complex binds to NAD^+^ and deacetylates the substrate protein. Recently, it has been reported that SIRT3 can reduce mitochondrial damage induced by oxidative stress through its deacetylation function, which helps stabilize the mitochondria [22]. The main three specific regulating mechanisms are:

(I) SIRT3 can increase ATP production and reduce ROS accumulation by regulating the activity of various important enzymes in the energy metabolism pathways in the mitochondria. Studies have shown that SIRT3 activates long-chain acyl-CoA dehydrogenase (LCAD) in fatty acid β-oxidation, to regulate the lipid metabolism balance [23] However, SIRT3 also promotes energy generation by regulating several key metabolism molecules, such as isocitrate dehydrogenase 2 (IDH2) in the tricarboxylic acid cycle [24], succinate dehydrogenase (SDH) of complex II [25], NADH dehydrogenase and ATP synthase [26]. It also activates acetyl-CoA synthetase 2 (AceCS2) by deacetylation, which promotes the formation of acetyl-coA [23,27]. In addition, SIRT3 also activates LKB1-AMPK pathway to increase ATP production [28].
(II) SIRT3 improves ROS removal from the mitochondria by enhancing the activity of various enzymes in the antioxidant system. Previously, studies have suggested that SIRT3 can increase the expression of manganese superoxide dismutase (MnSOD) and CAT by promoting the transcription of forkhead box O3 (FOXO3a) [29]. It can also deacetylate MnSOD and peroxisome proliferator-activated receptor–gamma coactivator 1α (PGC1α) directly, thereby enhancing the activity of the downstream antioxidant proteins and improving the ability to remove mitochondrial ROS. Moreover, SIRT3 activates glutamate dehydrogenase (GDH) and IDH2 in amino acid metabolism through deacetylation, to promote NADPH production, which in turn provides H^+^ for the glutathione reduction reaction, eventually increasing ROS hydrolysis [30,31].
(III) SIRT3 promotes the steady state of the mitochondrial environment. It mediates the deacetylation of Cyclophilin D (CypD), a modulatory component of the mitochondrial permeability transition pore (mPTP), which inhibits mPTP opening and delays mitochondrial swelling [30]. Furthermore, SIRT3 prevented cell death by promoting the interaction of Ku70 and Bax, which decreased Bax transfer into the mitochondria from the cytoplasm [32]. In addition, SIRT3 activates PGC1α, which is the transcription co-activator and transcription factor of many nuclear receptors. SIRT3 interacts with PGC1, resulting in acetylation, which plays an important role in mitochondrial DNA replication, transcription and protein synthesis [33].

## 4. Uncoupling Protein 2 (UCP2) and Cerebral Ischemia–Reperfusion Injury

Uncoupling protein (UCP), which is unique to mammals, exists in the mitochondrial inner membrane. It is composed of three u-shaped membrane units, each composed of 100 amino acids. Its C-terminal and N-terminal are located in the outer mitochondrial membrane, thus, UCP contains four segments in the cytosol and three segments in the mitochondrial matrix. Some of the amino acid residues in these segments form functional determinants. UCP2 is a new member of the mitochondrial uncoupling protein family. It is located on chromosome 11, which is highly conserved. UCP2 is widely distributed in tissues and organs, such as brown adipose tissue (BAT), white adipose tissue (WAT), and heart and intestinal mucosa [34,35]. In recent years, many studies have provided evidence that UCP2 plays a key role in basal metabolic rate regulation, oxidative stress response, insulin resistance and other pathological and physiological activities [36,37].

It has been shown that UCP2 is an important mitochondrial proton transporter. It can function as a proton channel, enabling the H^+^ on the mitochondrial inner membrane to flow back through the channel, thereby dissipating the electrochemical proton gradient across the membrane, and thus uncoupling the electron transfer and ATP production, resulting in decreased ATP generation. [38]. Under ischemia and hypoxia conditions, the increased ROS level induces the expression of UCP2 to decrease the proton gradient, which in turn reduces ROS generation. Moreover, UCP2 can increase the NAD^+^/NADH ratio by reducing the production of ATP, which accelerates the substrate oxidation and reduces ROS produced during the redox reaction. This negative feedback loop participates in cell protection [39]. Recently, experiments on UCP2-overexpressing mice have shown that the neuronal damage caused by ischemia–reperfusion was significantly lower than that in wild type mice. Furthermore, the ROS level was obviously lower compared with wild type mice [40,41]. In addition, the expression of genes related to antioxidants was suppressed in UCP2-knockout mice during cerebral ischemia–reperfusion, which suggests that UCP2 may decrease brain damage by reducing oxidative stress during ischemia–reperfusion.

## 5. UCP2-NAD^+^/NADH-SIRT3 Signaling Pathway

NAD^+^/NADH is a necessary coenzyme for cell energy metabolism. It can be synthesized in two ways: de novo synthesis and remediation synthesis. Recent studies have indicated that NAD^+^ not only participates in ATP production, but also indirectly regulates a variety of metabolic pathways via several NAD^+^-dependent proteins. These proteins have important roles in signal transduction, energy metabolism and mitochondrial adaptation. Among them, NAD^+^-dependent deacetylase SIRT3 is a key regulatory protein, which is able to sense the NAD^+^ levels (NAD^+^/NADH ratio). While NAD^+^ levels increase, the deacetylase activity of SIRT3 increases [42,43,44]. Investigators have shown that adding exogenous NAD^+^ to glomerular mesangial cells incubated with high glucose maintained the intracellular NAD^+^/NADH ratio and activated the SIRT3-AMPK-mTOR pathway, which blocks glomerular mesangial hypertrophy [45]. Another experiment demonstrated that the NAD^+^/NADH ratio and SIRT3 activity decreased in conditional knockout mice with a complex I defect, resulting in increased protein acetylation, which accelerates the progress of heart failure [46].

UCP2 improves the mitochondrial NAD^+^/NADH ratio by suppressing the ATP and ROS generation, and the NAD^+^ levels directly control the SIRT3 activity. Therefore, it is reasonable to speculate that UCP2 protects the mitochondria by increasing the NAD^+^/NADH ratio to activate SIRT3 in ischemia and hypoxia conditions.

However, we have to admit that the indirect link via the NAD^+^/NADH ratio between UCP2 and SIRT3 is not a compelling argument, since many metabolic factors (e.g. substrate supply) affect this ratio. Thus more evidence is needed to prove or disprove the UCP2-NAD+/NADH-SIRT3 signaling pathway and the role it plays in CIR.

## 6. Peroxisome Proliferator-Activated Receptor Gamma Coactivator 1-Alpha (PGC1α) Mediated Mitochondrial Biosynthesis

It is widely recognized that SIRT3 participates in mitochondrial protection. One of the important mechanisms is that SIRT3 affects mitochondrial biosynthesis by regulating the downstream antioxidant, peroxisome proliferator-activated receptor gamma coactivator 1-alpha (PGC1α), to reduce cell oxidative damage and improve mitochondrial function. This has been demonstrated in various diseases such as cardiac disease and cancer [47,48].

Human PGC1α is located in chromosome 4p15.1. PGC1α has two key characteristics: it is expressed in specific tissues and is induced by specific signals. The first means that PGC1 mainly exists in tissues that are rich in mitochondria or have a high energy demand, such as BAT, heart, skeletal muscles, kidney and brain. The second means that PGC1α is regulated by signals involved in metabolic demand sensing [48].

Mitochondrial biosynthesis is a physiological activity that maintains and repairs the mitochondrial structure, and is an important bridge for regulating mitochondrial genes and proteins, such as formatting the bilayer membrane of organelles, importing mitochondrial proteins encoded by nuclear genes, synthesizing the components of the electron transport chain in the mitochondrial matrix, and translating and replicating the mitochondrial genome. Recently, it has been suggested that the PGC1 family is the most important auxiliary transcription factor in mitochondrial biosynthesis [49]. Hence, the PGC1α-mediated nuclear transcription model affects mitochondrial biosynthesis and function.

Studies have indicated that PGC1α is particularly sensitive to cell energy, thus it can function as a sensor of metabolic stress or oxidative damage. Furthermore, PGC1α affects mitochondrial respiration, fatty acid metabolism and the ROS defense system by interacting with nuclear transcription factors (NRF1, NRF2 and ERRα) that prevent cell death [50,51]. On the one hand, NRF1 binds to most of the promoter regions of the complex I–V nuclear genes to promote gene transcription. One the other hand, NRF1 binds and activates the homologous mitochondrial transcription factors, TFAM and TFB, which promote mitochondrial DNA transcription and regulate mitochondrial genome replication and translation [52]. Recently, several investigators have indicated that ERRα participated in gene transcription involved in fatty acid oxidation. However, there are many studies suggesting that ERRα also regulates TFAM and ATP synthase α-subunits via PGC1α [53]. PGC1α may integrate mitochondrial biosynthesis signaling by forming different functional transcription activation complexes with NRF1, NRF2 and ERRα.

PGC1α also functions as an important intracellular antioxidant protein. Previous studies have shown that overexpressed PGC1α effectively suppressed ROS production and apoptosis caused by oxidative stress in endothelial cells [54]. In turn, suppressed PGC1α resulted in ROS accumulation, causing brain damage in cerebral ischemia [55].

## 7. Conclusions and Further Perspectives

The mitochondrion is the center of energy metabolism and many cellular events. Numerous studies have shown that timely and effective mitochondrial protection is a useful strategy in cerebral ischemia therapy. Herein, we suggest that UCP2 can improve the NAD^+^/NADH ratio, which activates the SIRT3 downstream target signaling molecule, PGC1α, to maintain mitochondrial biosynthesis and reduce damage caused by oxidative stress during cerebral ischemia–reperfusion injury (Figure 1B). However, there is still no clinical evidence that the brain protection mechanism of SIRT3 is mediated by UCP2. Thus, further research is needed to explain this potential pathway in cerebral ischemia–reperfusion injury.
